# GPR35 antagonist CID-2745687 attenuates anchorage-independent cell growth by inhibiting YAP/TAZ activity in colorectal cancer cells

**DOI:** 10.3389/fphar.2023.1126119

**Published:** 2023-04-11

**Authors:** Wuxiyar Otkur, Xiaolong Liu, Huan Chen, Siyi Li, Ting Ling, Hanchen Lin, Renyu Yang, Tian Xia, Huan Qi, Hai-Long Piao

**Affiliations:** Dalian Institute of Chemical Physics, Chinese Academy of Sciences, Dalian, China

**Keywords:** GPR35, colorectal cancer, YAP/TAZ, anchorage-independent growth, antagonist

## Abstract

**Background and purpose:** GPR35, a member of the orphan G-protein-coupled receptor, was recently implicated in colorectal cancer (CRC). However, whether targeting GPR35 by antagonists can inhibit its pro-cancer role has yet to be answered.

**Experimental approach:** We applied antagonist CID-2745687 (CID) in established GPR35 overexpressing and knock-down CRC cell lines to understand its anti-cell proliferation property and the underlying mechanism.

**Key results:** Although GPR35 did not promote cell proliferation in 2D conditions, it promoted anchorage-independent growth in soft-agar, which was reduced by GPR35 knock-down and CID treatment. Furthermore, YAP/TAZ target genes were expressed relatively higher in GPR35 overexpressed cells and lower in GPR35 knock-down cells. YAP/TAZ activity is required for anchorage-independent growth of CRC cells. By detecting YAP/TAZ target genes, performing TEAD4 luciferase reporter assay, and examining YAP phosphorylation and TAZ protein expression level, we found YAP/TAZ activity is positively correlated to GPR35 expression level, which CID disrupted in GPR35 overexpressed cells, but not in GPR35 knock-down cells. Intriguingly, GPR35 agonists did not promote YAP/TAZ activity but ameliorated CID’s inhibitory effect; GPR35-promoted YAP/TAZ activity was only partly attenuated by ROCK1/2 inhibitor.

**Conclusion and implications:** GPR35 promoted YAP/TAZ activity partly through Rho-GTPase with its agonist-independent constitutive activity, and CID exhibited its inhibitory effect. GPR35 antagonists are promising anti-cancer agents that target hyperactivation and overexpression of YAP/TAZ in CRC.

## 1 Introduction

G protein-coupled receptors (GPCRs) are the largest superfamily of transmembrane proteins in the human genome, with over 800 members mediating a wide range of signaling processes (Lagerström and Schiöth, 2008). Overexpression (Julius et al., 1989), mutation (Allen et al., 1991) of GPCRs, and hyper level of its endogenous ligands (Gutkind et al., 1991) can induce cancer cell-like transformation of NIH3T3 cells. These patterns were also observed in numerous cancer types in clinics (Dorsam and Gutkind, 2007), implying that targeting GPCR is promising in anti-cancer therapy (Soond and Zamyatnin, 2020). Throughout history, GPCRs have been the most “drugable” targets, accounting for approximately 35% of the targets of Food and Drug Administration (FDA)-approved drugs (Sriram and Insel, 2018). Currently, the development of GPCR targeting anti-cancer drugs is also prevalent (Usman et al., 2020).

GPR35, a member of the G protein-coupled receptors family, is considered an orphan receptor whose precise physiological function and endogenous ligand are under intensive investigation (De Giovanni et al., 2022; Sun et al., 2022; Wyant et al., 2022). GPR35 was found to be implicated in cancer development by numerous studies, especially in colon cancer (Ali et al., 2019; Hao et al., 2022; Mackiewicz et al., 2022), but the underlying mechanism is unclear. Recently, a constitutive ligand-independent pro-cancer function of GPR35 was reported, demonstrating that GPR35 interacted with Na^+^/K^+^-ATPase inducing colorectal cancer (CRC) cell proliferation and glycolysis (Schneditz et al., 2019), also driving macrophages to promote angiogenesis in the tumor microenvironment (Pagano et al., 2021).

Anchorage-independent growth occurs upon cell detachment from the correct extracellular matrix (ECM) and regrowth in the new metastatic site, a hallmark phenotype of cancer cell malignancy (Strilic and Offermanns, 2017; Hamidi and Ivaska, 2018). Here we investigated the cancer-promoting role of overexpressed GPR35 by performing two-dimensional (2D) culture conditions and tree-dimensional (3D) soft-agar anchorage-independent growth of CRC cells.

YAP and TAZ are two components in the Hippo pathway acting as transcriptional coregulators that promote the transcription of genes involved in cellular proliferation and suppress apoptotic genes, which assists anchorage-independent cell growth. Their dysregulation is intensively involved in cancer development, including CRC (Della Chiara et al., 2021). YAP/TAZ is predominantly regulated by mechano-transduction and also by GPCRs (Yu et al., 2012). Targeting GPCRs to regulate YAP/TAZ activity is a potential therapeutic strategy (Park and Guan, 2013) but is still underdeveloped. Whether GPR35 mediates YAP/TAZ activity has yet to be investigated.

Although the significant relevance of GPR35 in cancer progression has been gradually acknowledged, whether GPR35 antagonist is a potential anti-CRC agent has not been investigated yet. Therefore, we sought to understand whether the GPR35 antagonist promised to be developed as an anti-CRC agent by examining the effect of GPR35 antagonist CID-2745687 (CID) (Zhao et al., 2010) on anchorage-independent cell growth and further identifying the relevant signaling pathway.

## 2 Materials and method

### 2.1 Reagents

Zaprinast was obtained from Sigma Chemical Co. (St Louis, MO, United States). Pamoic acid and CID-2745787 (CID) was purchased from Cayman Chemical (Ann Arbor, Michigan, United States). 5-Hydroxyindoleacetic acid (5HIAA) was purchased from Shanghai Acmec Biochemical Co., Ltd. (Shanghai, China). Y-27632 was purchased from ApexBio Technology (Huston, TX, United States). Verteporfin was purchased from Shanghai Macklin Biochemical Co., Ltd. (Shanghai, China). Pertussis toxin was purchased from KKL MED Inc. (Ashland, VA, United States). ML145 was obtained from Tocris Bioscience Co. (St. Louis, MO, United States). All compounds were dissolved in dimethyl sulfoxide (DMSO) and stock solutions. UltraPure™ LMP Agarose were purchased from Invitrogen (Carlsbad, CA, United States).

### 2.2 Cell culture

Human colorectal adenocarcinoma HT-29, LOVO, CACO2, LS174T, HCT116, SW1116, THC8307, and DLD1 cells were kindly gifted by Sun Yat-sen University; and human embryonic kidney 293FT cells were obtained from Thermo Fisher Scientific. THC8397, HCT116 and 293FT cells were cultured in Dulbecco’s Modified Eagle Medium (DMEM) manufactured by Dalian Meilun Biotechnology Co., Ltd. (Dalian, China). HT-29, LOVO, CACO2, LS174T, SW1116, and DLD1 were culture with Roswell Park Memorial Institute-1640 Medium (RPMI-1640) manufactured by Dalian Meilun Biotechnology Co., Ltd. (Dalian, China). Culture mediums were supplemented with 10% certified fetal bovine serum, 100 μg/mL of streptomycin and 100 U/mL of penicillin. Cells were incubated at 37°C with 5% CO_2_ in a humidified atmosphere.

### 2.3 2D and 3D colony formation assay and cell detachment culture

For 2D colony formation assay, 200 cells were seeded in 6-well plates. Colonies were cultured for 14 days, medium were refreshed every 3 days. After then, cells were washed with PBS and stained with crystal violet solution for 30 min, and wash with ice-old water 3 times, 10 min for each. Colony were observed with phase-contract microscope, recorded by Tanon 1600 Automatic Digital Gel Image Analysis System produced by Shanghai Tanon Technology Co., Ltd. (Shanghai, China). The colony numbers and sizes were analyzed with TanonImage Software and ImageJ.

For 3D soft agar colony formation assay performed in 6-well plates, 5.0 × 10^3^ cells were seeded in upper phase, 0.35% agarose dissolved in DMEM or RPMI-1640 supplemented with 10% FBS, supported by 0.7% agarose dissolved in DMEM. Colony were cultured for 21 days, and stained with 0.5 mg/mL Nitrotetrazolium blue chloride purchased from Sangon Biotech Co., Ltd. (Shanghai, China). Soft agar formation under GPR35 antagonist treatment, 2.0 × 10^3^ cells were seeded in 12-well plates per well. The morphology, number and size of colonies were recorded and analyzed.

For detachment culture, cells were culture on 1% agarose coated plates at the density of 5.0 × 10^5^/mL, and same numbers of cells were cultured in normal culture plates as a parental control. After 48 h of culture, cells were harvested and subject to analysis.

### 2.4 Real-time PCR assay

Total cellular RNA was isolated by homogenizing tissue with IKA T10 basic ultra-turrax (Baden-Württemberg, Germany) and using RNAiso Plus from Takara Bio (Shiga, Japan). The yield and purity of the RNA were determined by spectroscopic analysis and the concentration of total RNA was equilibrated. cDNA was synthesized by using Evo M-MLV RT Kit by AG Accurate biology Co., Ltd. (Hunan, China). Finally, amplification of target genes was conducted by using SYBR Green Premix Pro Taq HS qPCR Kit AG Accurate biology Co., Ltd. (Hunan, China) in Bio-Rad CFX96TM Real-Time System (CA, United States). Primers used in present study were as follows: CTGF, 5′-CCA​ATG​ACA​ACG​CCT​CCT​G-3′, 5′-TGG​TGC​AGC​CAG​AAA​GCT​C-3’; CYR61, 5′- AGC​CTC​GCA​TCC​TAT​ACA​ACC-3′, 5′-TTC​TTT​CAC​AAG​GCG​GCA​CTC-3’; ANKRD1, 5′-AGT​AGA​GGA​ACT​GGT​CAC​TGG-3′, 5′- TGG​GCT​AGA​AGT​GTC​TTC​AGA​T-3’; G13, 5′- GTC​CAA​GGA​GAT​CGA​CAA​ATG​C -3′, 5′- AAG​TCC​TGC​CCG​TGG​ATG​AT-3’; Gi1, 5′- GGC​CAC​TGT​ACC​CAG​AGA​TTC -3′, 5′-CTA​AGC​GGA​GTC​GAG​GGA​GA’; ARB2, 5′- AAG​CTC​ACC​GTG​TAC​TTG​GG -3′, 5′-GGT​AGT​CAG​GGT​CCA​CAA​GC-3’; GPR35, 5′- GGA​CGC​CAT​CTG​CTA​CTA​CT-3′, 5′-GCA​CAG​AGA​GTC​CTG​GCT​TT-3’; GAPDH, 5′-CCA​TGT​TCG​TCA​TGG​GTG​TG-3′, 5′-CAG​GGG​TGC​TAA​GCA​GTT​GG-3’. All primers were purchased from Sangon Biotech Co., Ltd. (Shanghai, China). The expression level of the targeted genes in each sample was normalized with Ct value of Actin. The relative fold changes of expression to control group were presented.

### 2.5 Dual-luciferase reporter assay

293FT cells were seed in 12-well plates 2.0 × 10^5^ per well. After 24 h of culture, medium was replaced by fresh medium, and indicated volumes of medium containing GPR35 expression lentivirus were added. After 12 h of incubation, cells were transfected with 0.15 μg pGL4-TEAD4-luciferase and 0.05 μg pRL-TK. After 48 h of transfection, cells were harvested and subjected to Dula-Luciferase Reporter Assay System designed by Promega Biotech Co., Ltd. (WI, United States). Measurements were performed on a BioTek Cytation5 plate reader at room temperature.

### 2.6 Cell lines establishment and lentivirus infection

To establish GPR35 overexpressing cell lines, DLD1, THC8307 and HCT116 Cells were infected with GPR35 expression lentivirus, then cultured at least 3 passages to get GPR35-stably expressed cell lines. For target knocked-down cell lines, the cells were first infected with lentivirus. After 48 h of culture, 2 μg/mL puromycin were used to select stably knocked-down cell lines. shRNA oligos for G13, Gi1 and ARB2 knocked-down 293FT cell lines, and GPR35 knocked-down LS174T cell lines were as follow:

shG13, 5′-CCG​TGA​CGT​GAA​GGA​TAC​TAT-3′;

shGi1, 5′-GAG​GAG​TGT​AAA​CAA​TAC​AAA-3′;

shARB2, 5′-CTT​CGT​AGA​TCA​CCT​GGA​CAA-3′;

GPR35-5, 5′-GGA​CGC​CAT​CTG​CTA​CTA​CTA-3′;

GPR35-7, 5′-GCC​CTG​AAC​TCA​CTG​TGT​ATT-3′;

GPR35-8, 5′-AGG​AGC​ACC​CGG​CAC​AAT​TTC-3′;

GPR35-9, 5′-AGC​GAT​CAA​GCT​GGG​CTT​CTA-3′.

Lentivirus were produced in 293FT cells. To obtain GPR35 expression lentivirus, 293FT were transfected with 5 μg of pbobi-GPR35 or pbobi-Vector plasmids combined with 2.5 μg pMDL, 1.5 μg pVSVg and 1 μg REV using polyethylenimine (PEI). Transfection mass ratio of plasmids to PEI was 1:3. For sh-RNA lentivirus, cells were transfected with 5 μg of pLKO carrying shRNA oligos or pLKO-Vector plasmids combined with 4.5 μg of psPAX2 and 0.5 μg of pVSVg using polyethylenimine (PEI). Lentivirus were harvested after 48 h of transfection, and viruses were stored in −80°C for long term storage and 4°C for short term storage.

### 2.7 Western blot assay

For whole cell lysate, cells were wash with ice-cold PBS once, and were snap frozen with liquid nitrogen stored in −80°C, after the indicated treatments. Samples were lysed with RIPA lysis buffer (50 mM Tris-HCl pH 7.4, 150 mM NaCl, 0.5% deocycholate, 1% NP40, 0.1% SDS, and 1 mM EDTA) supplemented with proteinase inhibitor and phosphatase inhibitor cocktails, and subjected to SDS-PAGE electrophoresis after denaturation. Primary antibodies against YAP, TAZ, pLATS1 (s1079), Histone3, pYAP (s127), pYAP (s397), and PARP were purchased form Cell Signaling technology (MA, United States). Primary antibodies against GAPDH and β-Actin were purchased from Proteintech Group, Inc. (IL, United States). Peroxidase-conjugated secondary antibodies were purchased from Jackson ImmunoResearch (AB_2313567 and AB_10015289).

For nuclear and cytoplasmic isolation, cultured cells were washed with ice-cold PBS once. Then, hypotonic buffer solution (20 mM Tris-HCl pH7.4, 10 mM NaCl, 3 mM MgCl_2_) was added to suspension and transfer cells to tube. After 15 min of incubation, 10% NP40 was added to supernatants at the ratio of 1:20 and vertex followed by centrifugation. Supernatants were collected as cytoplasmic lysate. Precipitants were lysed with RIPA lysis buffer to isolate nuclear lysate. Nuclear and cytoplasmic lysate were subjected to SDS-PAGE electrophoresis after denaturation.

### 2.8 Data analysis

All experiments were biologically replicated at least three times. The data are presented as the means ± SDs and were statistically analyzed by using GraphPad Prism 8.0 software. One-way or two-way ANOVA followed by Tukey’s or Dunnett’s multiple comparisons test was used to evaluate the statistical significance among groups. For comparisons between two groups, unpaired t-tests were used. In soft agar formation assay, for comparisons between two groups, paired t-tests were used. A *p*-value <0.05 was considered to indicate statistical significance, *p* < 0.05, *; *p* < 0.01, **; *p* < 0.001, ***; *p* < 0.0001, ****.

## 3 Results

### 3.1 GPR35 promotes anchorage-independent growth in CRC cells

**FIGURE 1:**
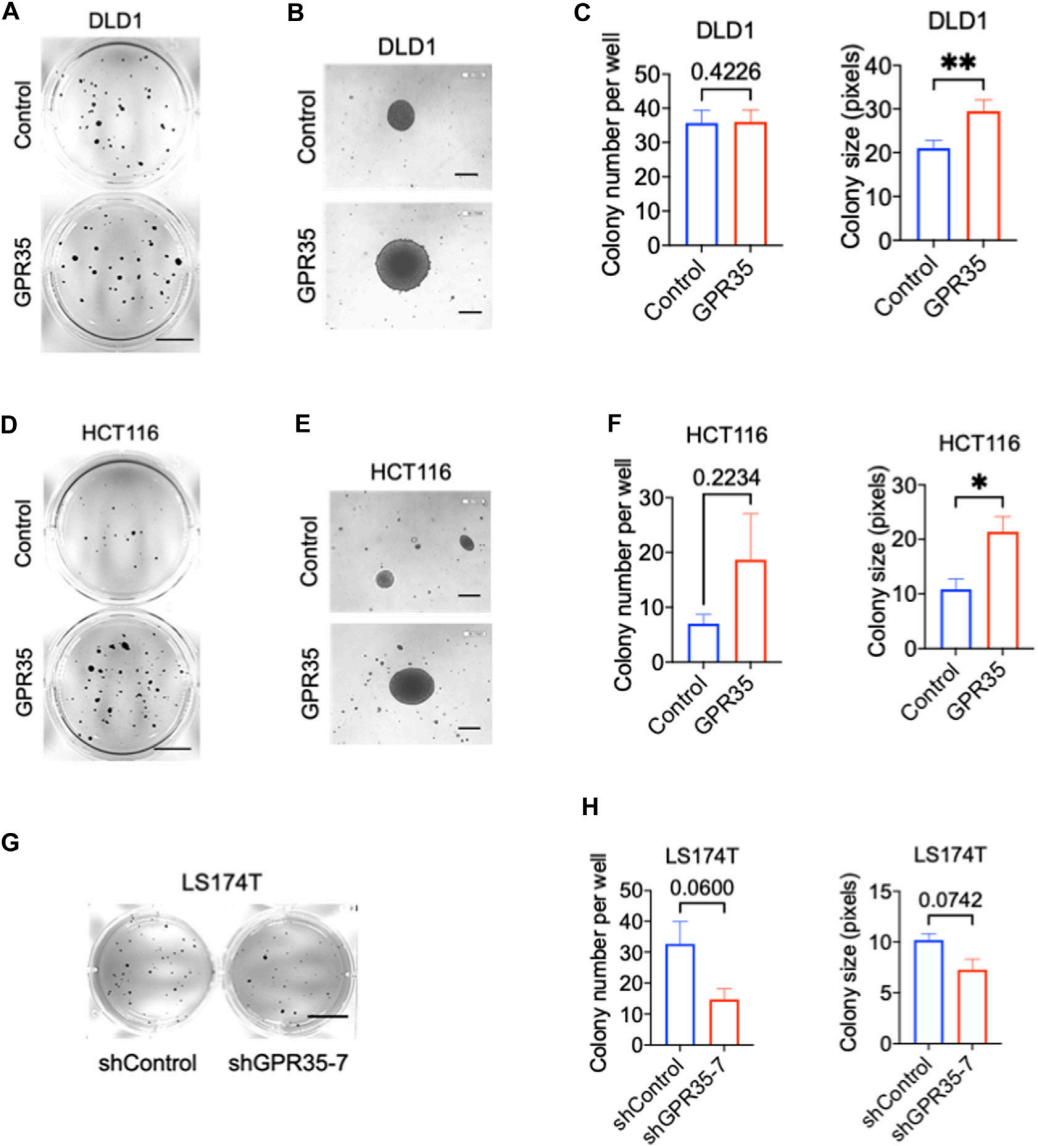
GPR35 promoted anchorage-independent growth in CRC cells. **(A–C)** Representative images of colonies formed by DLD1 cell lines in soft agar in 21 days and quantification data of soft agar colony formation assay (*n* = 3). Scale bars: 1.0 cm. **(B)** Colonies observed with a microscope. Scale bars: 0.1 mm. **(D–F)** Representative images of colonies formed by HCT116 cell lines in soft agar in 21 days, and quantification data of soft agar colony formation assay (*n* = 3). Scale bars: 1.0 cm. **(E)** Colonies observed with a microscope. Scale bars: 0.1 cm. **(G,H)** Representative images of colonies formed by LS174T cell lines in soft agar in 21 days, and quantification data of soft agar colony formation assay (*n* = 3). Scale bars: 1.0 cm.

Firstly, we examined the expression of GPR35 in different CRC cell lines. Among 8 different CRC cell lines, DLD1, THC8307, and HCT116 exhibited relatively low expression of GPR35 (Supplementary Figure S1A). To investigate the pro-cancer function of GPR35, we stably overexpressed GPR35 in DLD1, THC8307, and HCT116 cell lines (Supplementary Figure S1B). In parallel, since LS174T expressed the highest level of GPR35, we established GPR35 knocked-down LS174T cells using shRNA (Supplementary Figure S1C). Next, we conducted 2D and 3D soft-agar colony experiments on these GPR35 overexpressed CRC cell lines. The result indicated that 2D colony formation and cell proliferation showed inconsistent results in three GPR35 overexpressed cell lines (Supplementary Figure S2A). However, in soft-agar colony formation assay, GPR35 overexpression consistently promoted colony-forming ability, especially colony size (Figures 1A–F). Consistently, GPR35 knock-down reduced both colony number and size in LS174T cells (Figures 1G, H). Together, these results indicated that GPR35 played a significant role in the cell growth of detached cells.

### 3.2 CID-2745687 attenuates anchorage-independent growth in GPR35 expressing CRC cells

**FIGURE 2:**
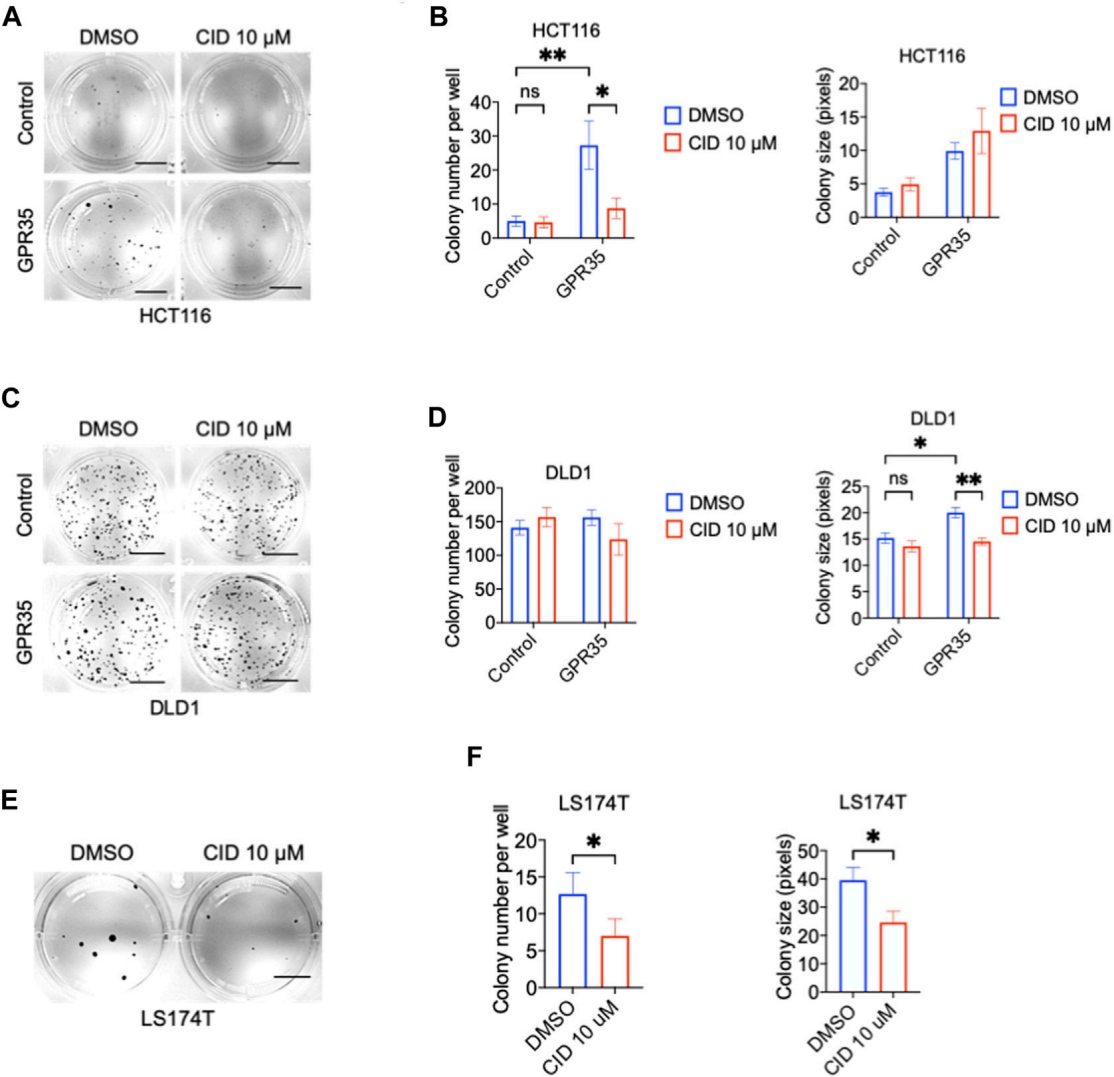
CID-2745687 attenuated anchorage-independent cell growth in GPR35-expressing CRC cells. **(A,B)** Representative images of colonies formed by HCT116 cell lines in soft agar in 21 days and quantification data of soft agar colony formation assay (*n* = 3). Scale bars: 0.5 cm. **(C,D)** Representative images of colonies formed by DLD1 cell lines in soft agar in 2 days, and quantification data of soft agar colony formation assay (*n* = 3). Scale bars: 0.5 cm. **(E,F)** Representative images of colonies formed by LS174T cell lines in soft agar in 21 days, and quantification data of soft agar colony formation assay (*n* = 3). Scale bars: 0.5 cm. Cells were suspended in a medium with 10 μM CID and seeded in soft agar. The medium was refreshed, and 10 μM of CID was added every 3 days.

Next, we tested whether GPR35 antagonist CID exhibited an inhibitory effect on colony formation in CRC cells. CID significantly inhibited GPR35-promoted colony formation at the concentration of 10 μM (Figures 2A–D). In line with this, CID treatment also decreased colony number and size in GPR35 expressing LS174T cells (Figures 2E, F). These results indicated that GPR35 antagonist CID inhibited the role of GPR35 played in anchorage-independent growth.

### 3.3 GPR35 moderates the phosphorylation of YAP/TAZ and preserves YAP/TAZ activity

Although GPR35 overexpressed cells did not promote 2D colonies (Supplementary Figures S2A, B), they significantly altered cell morphology and formed denser and crowed-like colonies than control cells, especially in DLD1 and HCT116 cells (Supplementary Figures S2C, D). These phenomena implied that GPR35 overexpressing cells counteracted with space restriction and proliferated in high-density conditions. Besides, GPR35 promoted the growth in cell detachment, reminiscing the function of YAP/TAZ (Zhao et al., 2007; Zhao et al., 2012).

**FIGURE 3:**
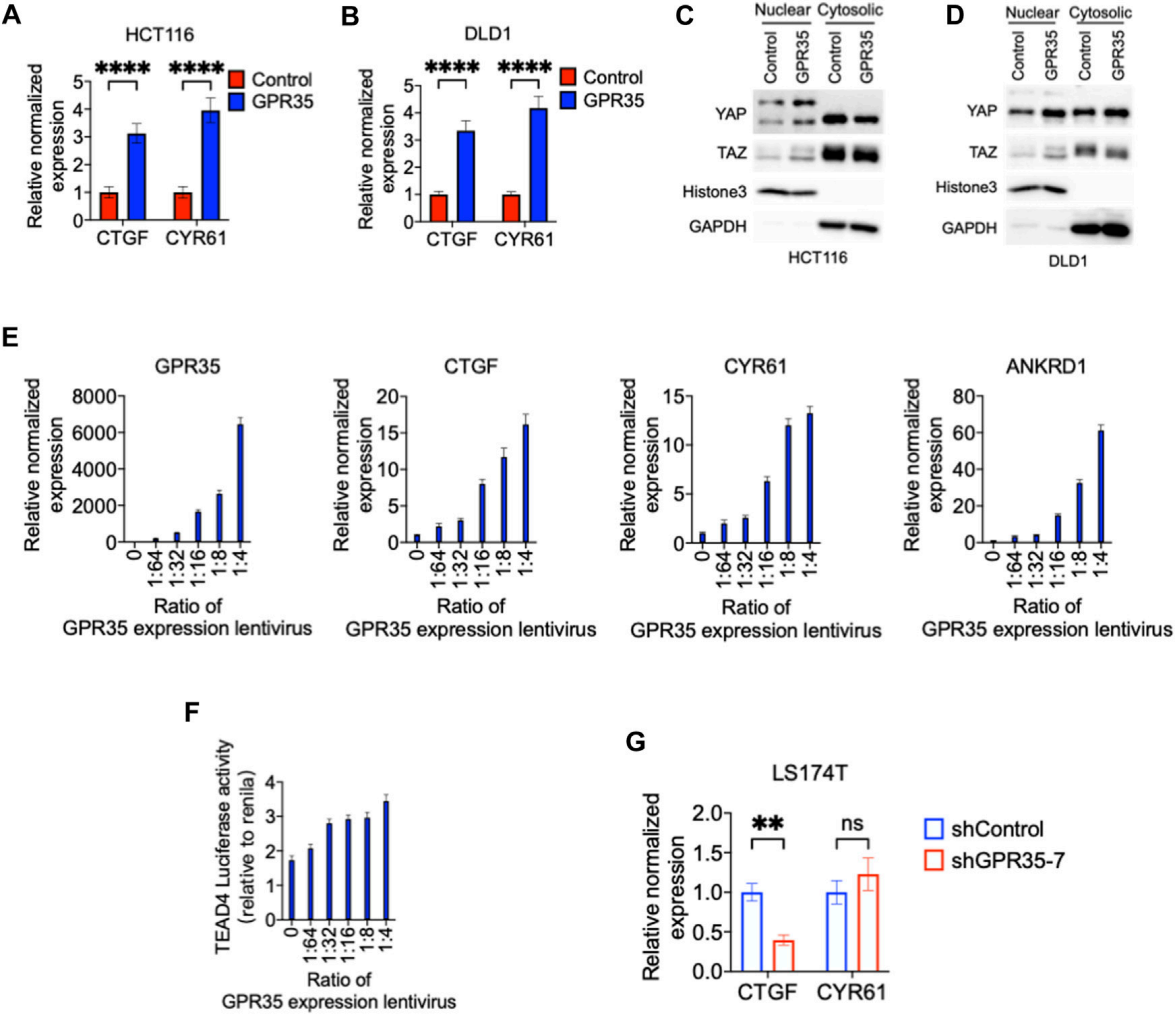
GPR35 expression enhanced YAP/TAZ activity. **(A,B)** Normalized mRNA expression of CTGF and CYR61 in established cell lines relative to parental control cell lines; **(C,D)** Nuclear and cytoplasmic distribution of YAP and TAZ analyzed by Western blot; **(E)** The normalized mRNA expression level of GPR35 relative to non-infected 293FT cells; and Normalized mRNA expression of CTGF, CYR61, and ANKRD1; Cells were infected with GPR35 expression lentivirus at the different ratios and harvested for real-time PCR assay after 48 h of incubation. **(F)** Dual-luciferase reporter assay on TEAD4 luciferase activity relative to Renilla luciferase activity. Cells harvest for the assay after 48 h of incubation with different concentrations of GPR35 expression lentivirus. **(G)** Normalized mRNA expression level of CTGF and CYR61 relative to shControl cells.

Therefore, we sought to understand the regulatory effect of GPR35 on YAP/TAZ activity. CTGF and CYR61, hallmark target genes of YAP/TAZ, were highly expressed in GPR35 overexpressing cells relative to control cells (Figures 3A, B) and indicated that GPR35 enhances YAP/TAZ expression in the nucleus (Figures 3C, D). Furthermore, GPR35 overexpressing cells formed a crow-like colony; we asked whether GPR35 attenuated the decrease of YAP/TAZ activity in high cell density conditions since high cell density condition downregulates YAP/TAZ activity and thus inhibits cell proliferation. Consistently, GPR35 overexpression ameliorated the downregulation of YAP/TAZ target gene expression in DLD1 and HCT116 cells (Supplementary Figures S3A, B).

To further confirm whether GPR35 modulate YAP/TAZ activation and whether GPR35 expression level is correlated to YAP/TAZ activity, we transfected 293FT cells with different amount of GPR35 expression lentivirus. First, the level of GPR35 expression was dose-dependently upregulated by lentivirus infection (Figure 3E). Subsequently, CTGF, CYR61, and ANKRD1 were upregulated dose-dependent manner by GPR35 expression (Figure 3E). Consistently, TEAD4 luciferase reporter assay also indicates that GPR35 dose-dependently upregulated YAP/TAZ transcriptional activity (Figure 3F). Consistently, knock-down of GPR35 significantly decreased CTGF expression, but not the expression of CYR61 in LS174T cells (Figure 3G). These indicated that GPR35 expression enhances YAP/TAZ activity.

### 3.4 GPR35 moderates the phosphorylation of YAP/TAZ

**FIGURE 4:**
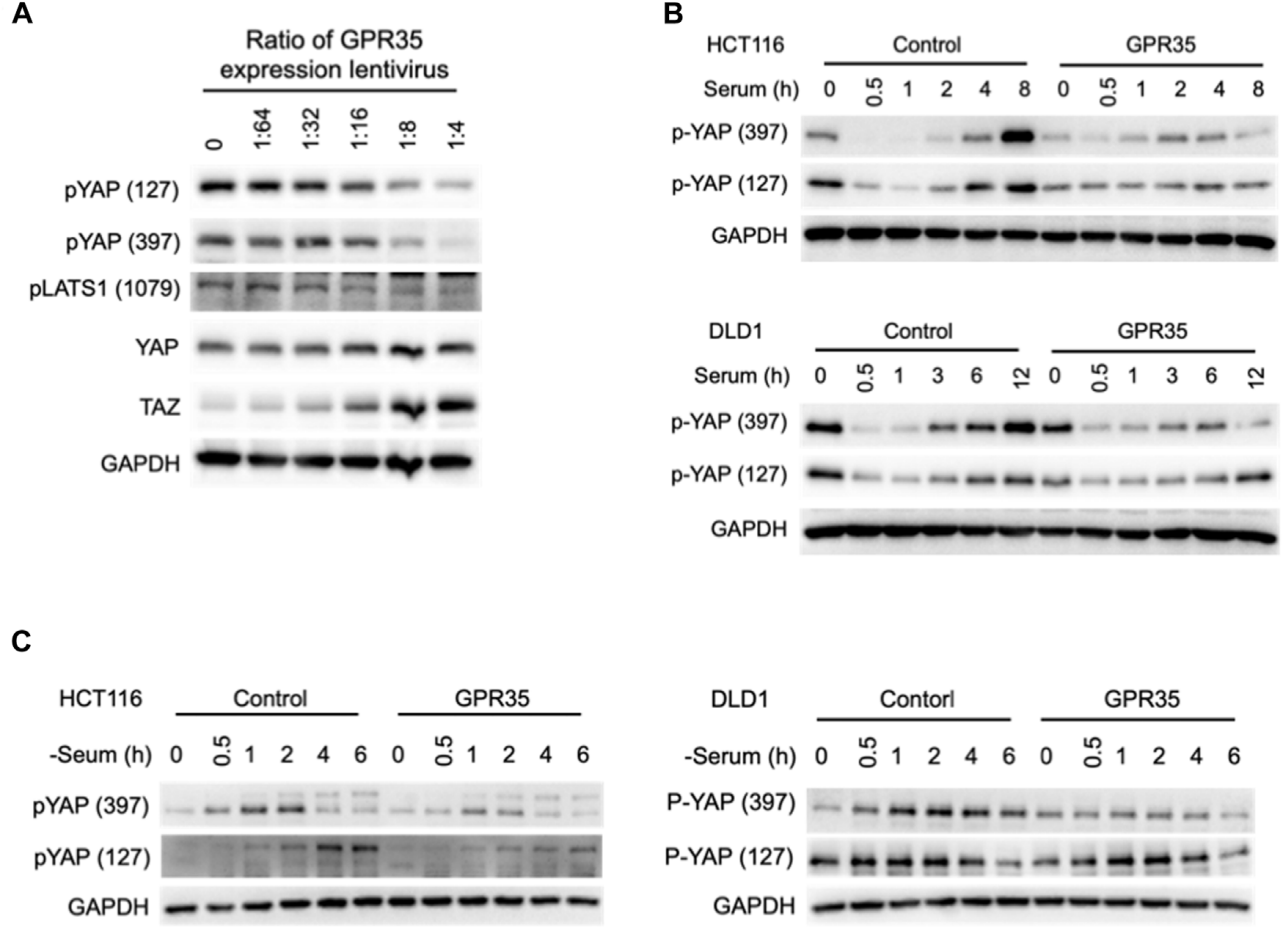
GPR35 expression moderated YAP/TAZ phosphorylation. **(A)** Western blot assay on YAP, TAZ, phosphorylated YAP, TAZ, and LATS1 after the indicated treatments. 293FT cells harvest for the assay after 48 h of incubation with different concentrations of GPR35 expression lentivirus. **(B)** Western blot analysis of phosphorylated YAP. After 24 h of culture, cells were subject to fresh medium containing 10% FBS. Cells were harvested at indicated time points; **(C)** Western blot assay on phosphorylated YAP after serum deprivation. After 24 h of culture, the medium was replaced by fresh serum-free medium and incubated for the indicated time.

YAP/TAZ is regulated by phosphorylation. In 293FT cells, the amount of GPR35 expression was negatively correlated with the phosphorylation level of YAP and its upstream kinase LAST1 and positively correlated with YAP and TAZ protein expression levels (Figure 4A).

To better understand GPR35 expression regulating YAP/TAZ activity, we detected the YAP phosphorylation after serum treatment and deprivation (Yu et al., 2012) in GPR35-expressing CRC cells. In control cells, serum treatment caused dephosphorylation of YAP within 1 h, and re-phosphorylated afterward (Figure 4B). However, in GPR35 overexpressing cells, the re-phosphorylation of YAP was suppressed (Figure 4B). Serum deprivation induced YAP phosphorylation in control cells, which is also attenuated in GPR35 overexpressing cells (Figure 4C). These provided that the expression level of GPR35 is positively correlated with the YAP/TAZ activity and negatively correlated with the activation Hippo pathway. GPR35 moderates YAP/TAZ phosphorylation and thus preserves their activities.

### 3.5 CID-2745687 inhibits GPR35-meditated YAP/TAZ activity

**FIGURE 5:**
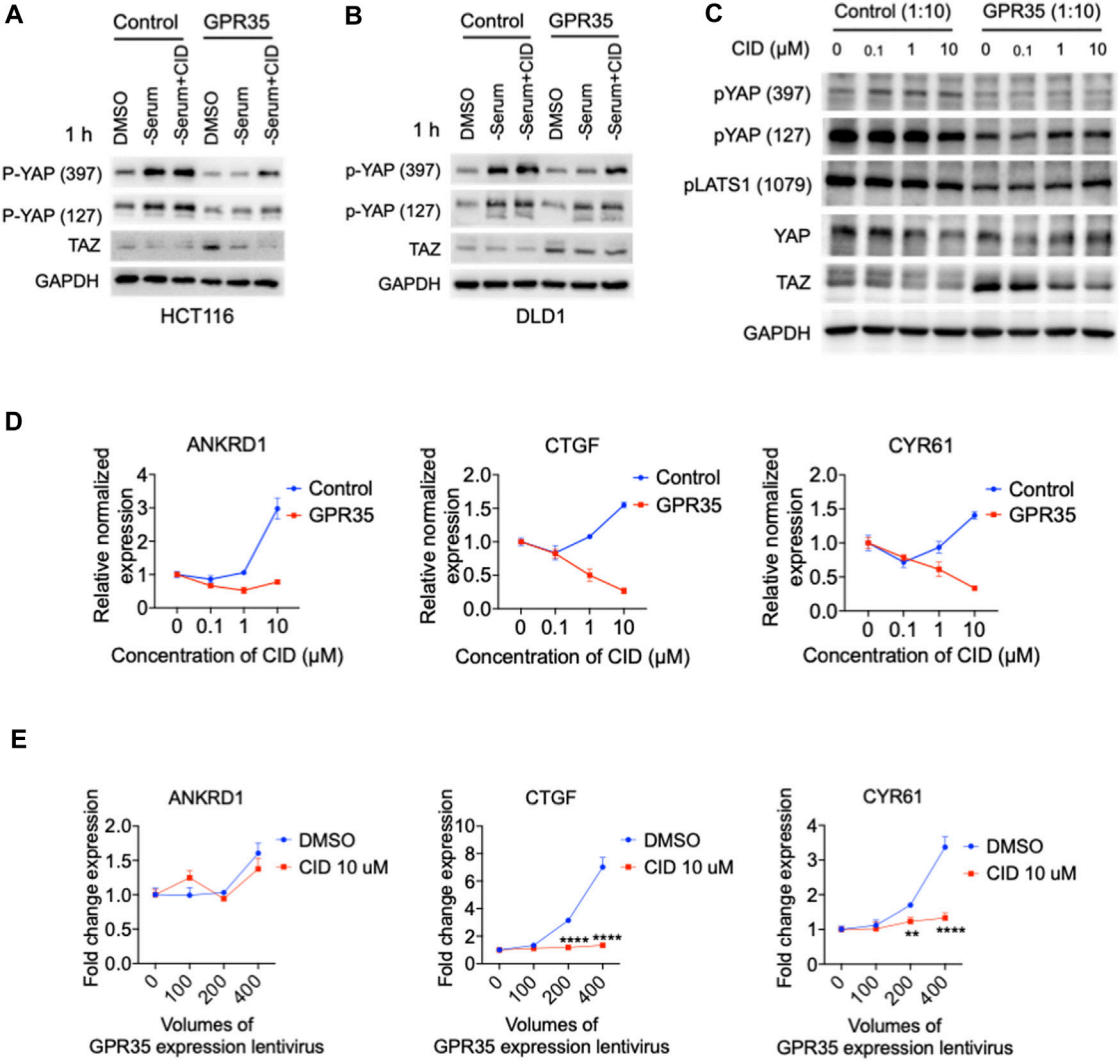
CID-2745687 inhibited GPR35-meditated YAP/TAZ activity. **(A,B)** Western blot assay on TAZ expression and YAP phosphorylation in established cell lines were challenged with serum deprivation for 1 h. Cells were subjected to fresh serum-free medium, and different CID concentrations were added simultaneously with serum-free medium. **(C)** Western blot analyzing expression and phosphorylation of YAP, TAZ, and LATS1 after treatment with different concentrations of CID. 293FT cells were infected with GPR35 expression lentivirus at the ratio of 1:10 for 24 h, and the different concentrations of antagonists were added directly to the medium. **(D)** mRNA expression of YAP/TAZ target genes after treatment of CID. **(E)** mRNA expression of YAP/TAZ target genes. 293FT cells were infected with different volumes of GPR35 expression lentivirus for 24 h, and CID was added directly to the medium for another 24 h. Then cells were harvested for PCR assay.

Next, we verified whether this GPR35-meditated YAP/TAZ activity could be regulated by its antagonist. In two different cell lines, GPR35 overexpression impaired serum deprivation-induced YAP phosphorylation compared to in control cells, the presence of CID rescued serum deprivation-induced YAP phosphorylation in GPR35 overexpressed CRC cell lines (Figures 5A, B).

In GPR35-transduced 293FT cells, we used two different GPR35 antagonists, ML145 (Heynen-Genel et al., 2010) and CID, to verify the inhibitory effect of GPR35 antagonist on YAP/TAZ activity. GPR35 antagonists decreased the expression level of YAP and TAZ (Figure 5C and Supplementary Figure S4A). CID downregulated YAP/TAZ target genes in a concentration-dependent manner in GPR35-expressing cells but not control cells (Figure 5D). Significantly, 10 μM of CID blocked the positive correlation between the expression level of GPR35 and YAP/TAZ target genes (Figure 5E). Similarly, ML145 also decreased YAP/TAZ target genes (Supplementary Figure S4B). However, CID and ML145 upregulated ANKRD1 expression in control cells by unknown mechanisms (Figure 3B and Supplementary Figure S5A). Results showed that CID is more potent than ML145 in regulating YAP/TAZ activity. These results indicated that the GPR35 antagonist effectively inhibits YAP/TAZ activity in GPR35 overexpressing cells.

### 3.6 GPR35 knock-down attenuated the inhibitory effect of CID-2745687 on YAP/TAZ activity

**FIGURE 6:**
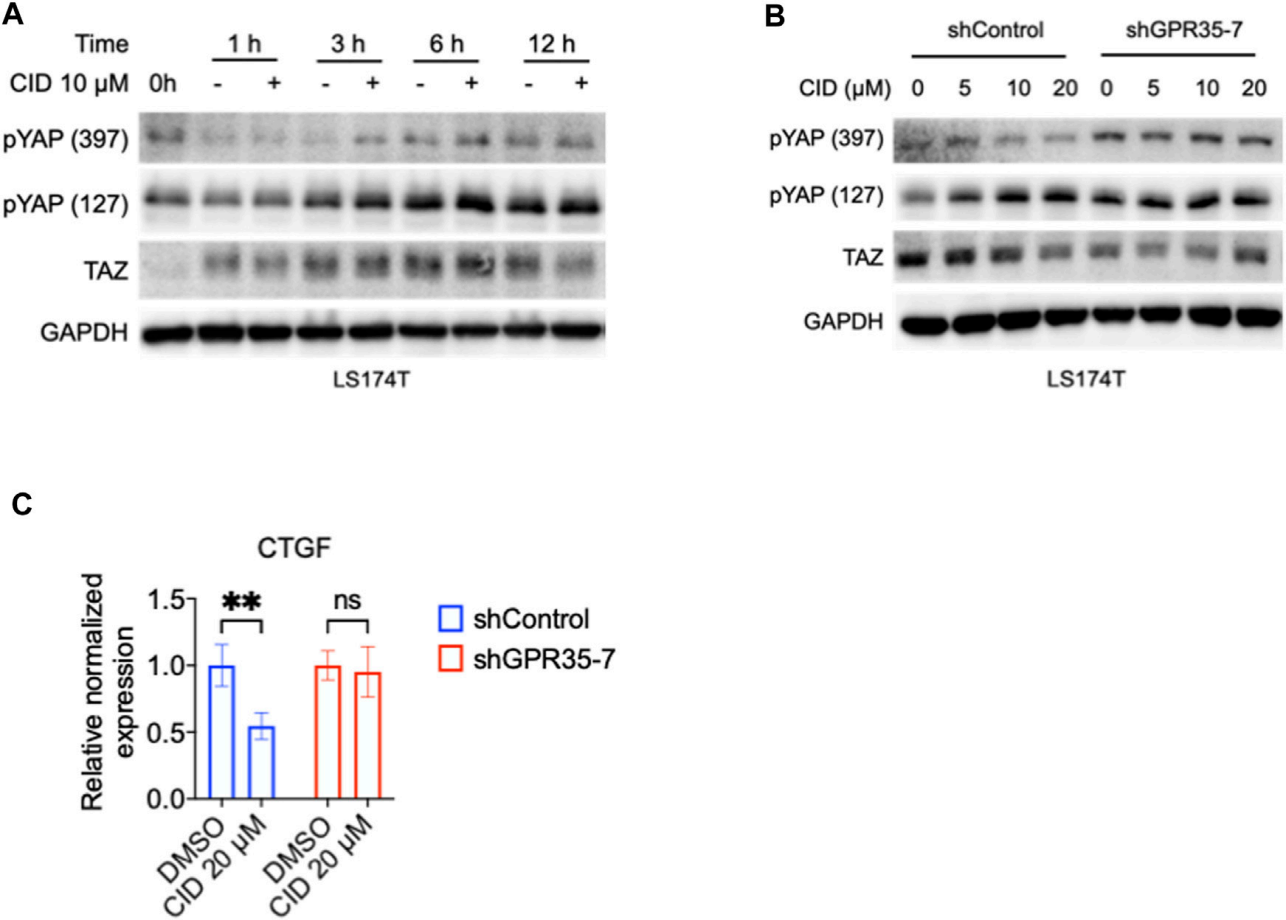
GPR35 knock-down attenuated the inhibitory effect of CID on YAP/TAZ activity. **(A)** Western blot assay on TAZ expression and YAP phosphorylation in LS174T cells. After 24 h of culture, cells were subject to a fresh medium containing 10% FBS with or without CID for the indicated time. **(B)** Western blot assay on TAZ expression and YAP phosphorylation in shControl and shGPR35-7 LS174T cells. After 24 h of culture, cells were subject to a fresh medium containing 10% FBS with or without CID for 3 h **(C)** mRNA expression of CTGF. Cells were harvested for analysis after 12 h of treatment.

To further confirm the effect of the antagonist on the GPR35-YAP axis, we tested the CID on GPR35 knock-down LS174T cells. We found that after 3 h of CID treatment, the phosphorylated YAP level significantly increased compared with vehicle treatment in LS174T cells (Figure 6A). Therefore, we examined the CID efficiency at 3 h of treatment in control and GPR35 knocked-down LS174T cells. YAP phosphorylation increased, and the TAZ’s expression decreased by CID dose-dependently in shControl cells. However, these responses were weakened in GPR35 knocked-down LS174T cells (Figure 6B). CID caused downregulation of CTGF was diminished in GPR35 knock-down LS174T cells (Figure 6C). These results indicated that the antagonist inhibited YAP/TAZ through GPR35.

### 3.7 GPR35 overexpression promotes anchorage-independent growth through YAP

**FIGURE 7:**
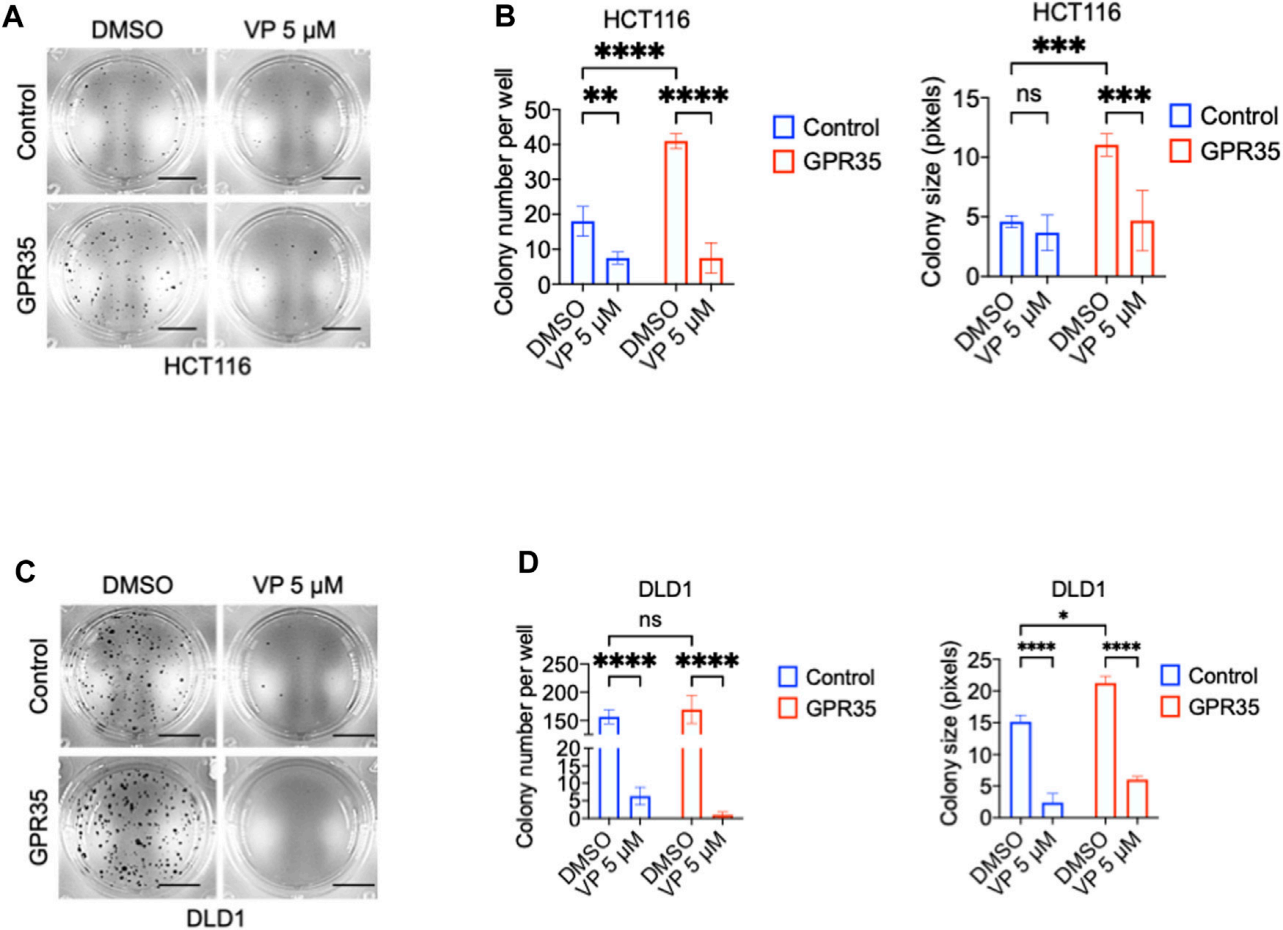
YAP/TAZ activity was required for anchorage-independent growth of CRC cells. **(A,B)** Representative microscopic images and quantification analysis of colony formation assay (*n* = 3) of HCT116 cell lines in soft agar assay. Scale bars: 0.5 cm. **(C,D)** Representative microscopic images and quantification analysis of colony formation assay (*n* = 3) of DLD1 cell lines in soft agar assay. Scale bars: 0.5 cm. Verteporfin 5 μM was used, and the culture medium was replaced every 3 days.

We questioned whether the pro-cancer phenotype exhibited by GPR35 overexpression depends on YAP activity. Consequently, we used the YAP/TAZ inhibitor verteporfin (VP) in soft-agar colony assay. Results showed that 5 μM of VP impaired the colony formation promoted by GPR35 overexpression (Figures 7A–D). However, VP showed potent inhibition in control cell lines. Therefore, YAP/TAZ activity is indispensable for CRC cell anchorage-independent growth. Furthermore, inhibition of GPR35-promoted YAP/TAZ activity by CID contributed to the impaired anchorage-independent growth in GPR35 overexpressed cell lines.

### 3.8 CID inhibits GPR35 agonists-independent constitutive activity on YAP/TAZ pathway

**FIGURE 8:**
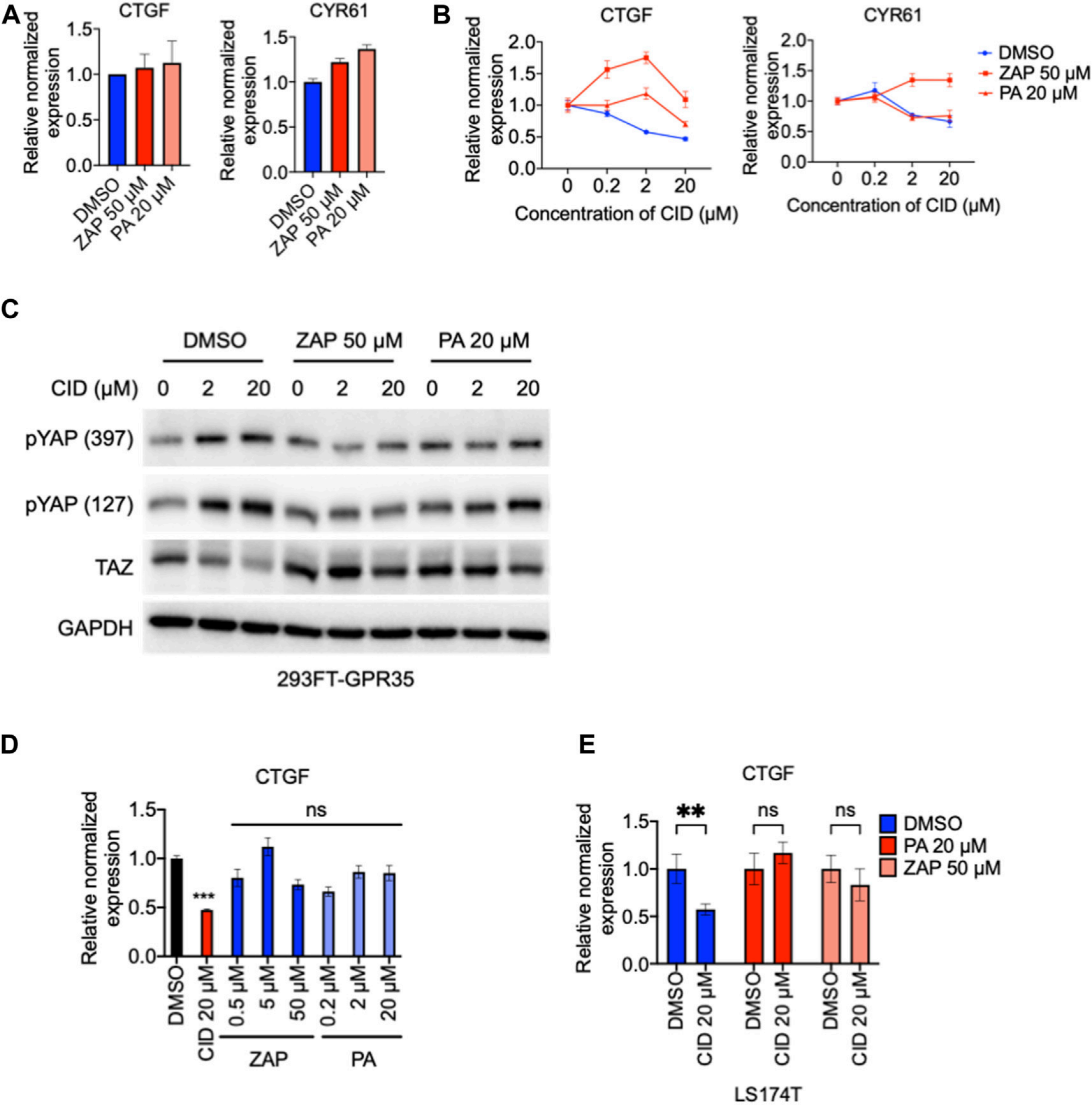
GPR35 agonists did not promote YAP/TAZ activity but attenuated the regulatory effect of CID-2745687. 293FT cells were infected with GPR35 expression lentivirus at different ratios of 1:20 for 24 h, and 50 μM zaprinast or 20 μM pamoic acid were added directly to the medium. **(A)** mRNA expression of YAP/TAZ target genes after treatment of 50 μM zaprinast or 20 μM pamoic acid. **(B)** mRNA expression of YAP/TAZ target genes after treatment of pamoic acid combined with different concentrations of CID. **(C)** Western blot analysis of YAP phosphorylation and TAZ expression. 293FT cells were infected with GPR35 expression lentivirus at different ratios of 1:10 for 24 h, and indicated concentrations of agonists were added directly to the medium. After 1 h of incubation, different concentrations of CID were added. Cells were harvested for analysis after 24 h of drug treatment. **(D)** mRNA expression of CTGF, LS174T cells were exposed to indicated treatments for 12 h, and harvested for analysis. **(E)** mRNA expression of CTGF and CYR61 in LS174T cells after 12 h of agonists treatments with or without 20 μM of CID.

GPR35 functions in agonist-dependent activity and agonist-independent constitutive activity (Schneditz et al., 2019; Pagano et al., 2021). Here, to understand how GPR35 promotes YAP/TAZ activity, we compare the YAP/TAZ responses to GPR35 agonists, zaprinast (Taniguchi et al., 2006), and pamoic acid (Zhao et al., 2010) in control and GPR35 overexpressed cells. Results found that the responsive kinetics in both cell lines were similar to both agonists (Supplementary Figure S5). Both zaprinast and pamoic acid did not increase the expression level of YAP/TAZ target genes (Figure 8A). These results indicated that GPR35 promotes YAP/TAZ activity through its agonist-independent constitutive activity.

Next, we examined whether GPR35 allosteric modulation by agonists influences the inhibitory effect of CID on YAP/TAZ activity. The results showed that although agonists did not promote YAP/TAZ target genes in GPR35 overexpressed 293FT cells (Figure 8A), attenuated the inhibitory effect of CID on YAP/TAZ target genes expression, especially the widely accepted zaprinast (Figure 8B). Pamoic acid moderated the effect on CID action (Figure 8B). Consistently, similar results were observed in LS174T cells. Furthermore, although agonists did not promote YAP/TAZ target genes expression (Figure 8C), they attenuated CID-induced downregulation of CTGF expression (Figure 8D). Western blot showed that CID dose-dependently increased YAP phosphorylation and decreased TAZ expression. These were ameliorated by zaprinast and pamoic acid pretreatments (Figure 8E).

These data indicated that GPR35 promotes YAP/TAZ activity with its agonist-independent constitutive activity. Furthermore, these also demonstrated that CID works through perturbing GPR35 constitutive activity.

### 3.9 GPR35 promotes YAP/TAZ activity partly through Rho-GTPase

**FIGURE 9:**
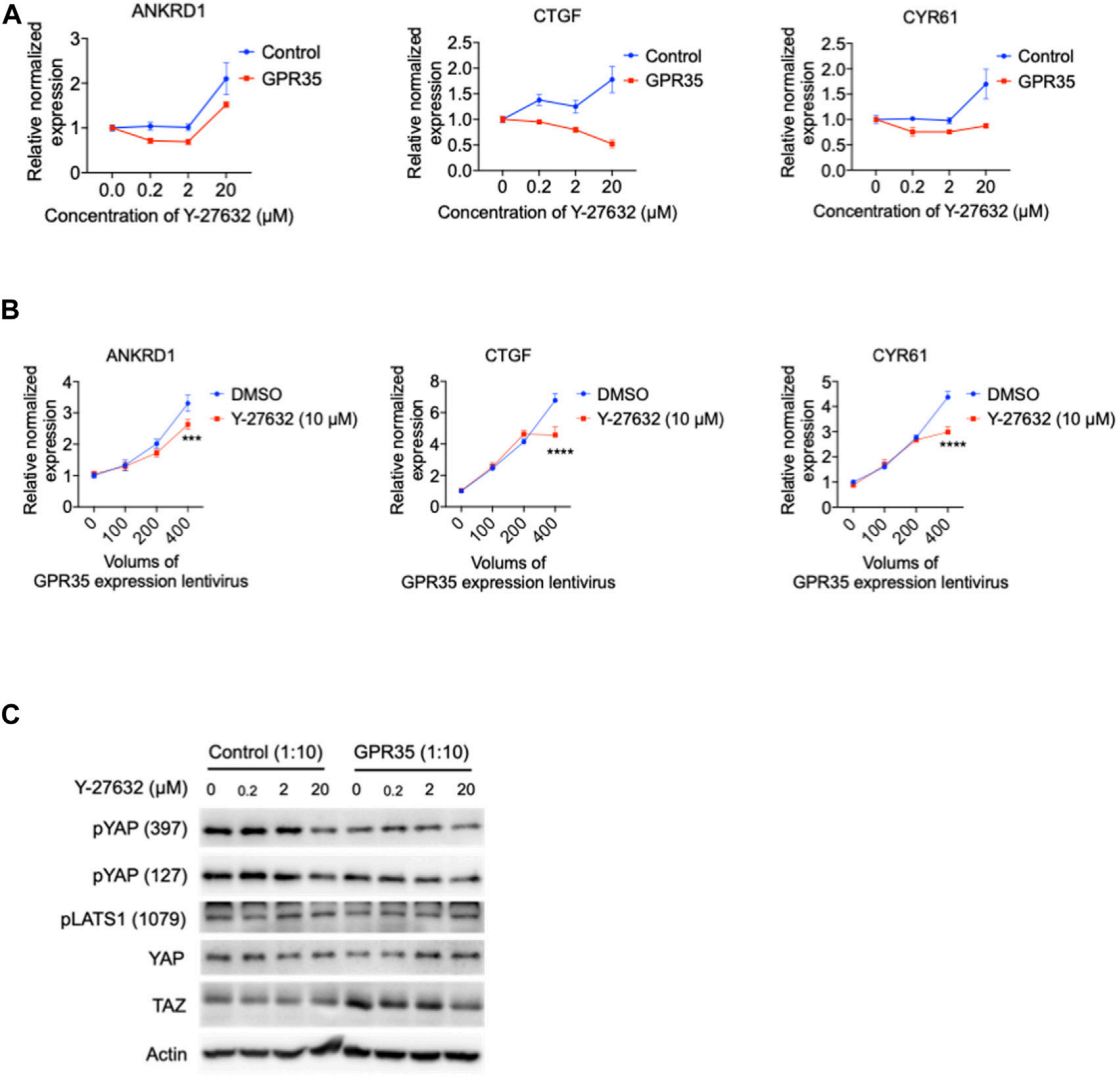
ROCK1 kinase inhibitor Y-27632 partly inhibited GPR35-mediated YAP/TAZ activity. 293FT cells were infected with GPR35 expression lentivirus at different ratios of 1:10 for 24 h, and different concentrations of Y-27632 were added directly to the medium. **(A)** mRNA expression level of YAP/TAZ target genes. **(B)** The mRNA expression of YAP/TAZ target genes. 293FT cells were infected with different volumes of GPR35 expression lentivirus for 24 h, and 10 μM Y-27632 was added directly to the medium for another 24 h. Then cells were harvested for PCR assay. **(C)** Western blot assay on YAP and TAZ expression levels, phosphorylation levels, and LATS1 phosphorylation levels.

GPCR regulates the Hippo pathway through a small GTPase protein known as Ras homolog family member A (RhoA) (Yu et al., 2012), activating ROCK1 to modulate the Hippo pathway. RhoA/ROCK1 is also reported to associate with GPR35 (McCallum et al., 2015). A specific RhoA/ROCK1 inhibitor Y-27632 was examined in GPR35 overexpressed 293FT cells. Y-27632 dose-dependently downregulated CTGF expression in GPR35 overexpressed cells (Figure 9A) and suppressed the expression of ANKRD1 and CYR61 at a concentration within 2 μM (Figure 9A). Next, we further tested the inhibitory effect of Y-27632 on the correlation of YAP/TAZ activity with the upregulation of GPR35 expression. Y-27632 of 10 μM only partly suppressed YAP/TAZ target genes expression induced by the highest level of GPR35 expression (Figure 9B). It is different from the effect shown by CID (Figure 5E). Western blot showed that the concentration of 20 μM of Y-27632 suppressed TAZ upregulation by GPR35 expression (Figure 9C). Nevertheless, none of these YAP/TAZ target genes were decreased in control cells (Figure 9). These indicated that GPR35 promoted YAP/TAZ partly by inducing RhoA/ROCK1 activation.

## 4 Discussion

In the present study, we demonstrated that GPR35 overexpression can promotes anchorage-independent cell growth and YAP/TAZ activity, and this pro-cancer role of constitutive GPR35 activity can be effectively inhibited by its antagonist.

GPR35 was considered a marker of aggressive tumor cells since it was detected to be expressed at a higher level in a regional lymph node in colon cancer patients (Ali et al., 2019). Usually, cells detached from the correct ECM compromise their proliferation and proceed into anoikis. Only the most aggressive tumor cells would survive, metastasize, proliferate, and colonize in a new microenvironment (Strilic and Offermanns, 2017; Hamidi and Ivaska, 2018). Our findings suggested that upregulation of GPR35 expression can possibly assist those aggressive tumor cells to successfully metastasize by promoting anchorage-independent cell growth and even inhibiting anoikis (Supplementary Figures S6A–C). Here, we provide additive evidence to charge GPR35, a marker of aggressive tumor cells. GPR35 is known to be expressed in two isoforms. It was GPR35b that is highly expressed in primary and metastatic tumor cells (Okumura et al., 2004; Ali et al., 2019) and exhibited higher constitutive activity (Schihada et al., 2022). We only focused on the function of the canonical GPR35a isoform in the present work. In the future, it would be necessary to investigate whether GPR35b is more potent in mediating YAP/TAZ activity.

More importantly, CID showed an inhibitory effect on GPR35-mediated tumor cell malignant phenotype. In the present study, GPR35 antagonist CID exhibited specific activity inhibiting anchorage-independent growth and promoting anoikis (Supplementary Figure S6D), showing auspicious properties in anti-CRC. However, the concentration required for this was relatively high, ranging in micromolar level. Therefore, future works can be done screening more active GPR35 antagonists for anti-cancer drug development. Furthermore, GPR35 only disclosed its cancer-promoting role in soft agar assay rather than in 2D plate culturing conditions. To be noted, it is necessary to identify the anti-cancer property and investigate the underlying mechanism of the GPR35 antagonist using 3D models. Moreover, GPR35 antagonists display high species ortholog selectivity (Jenkins et al., 2012), implying caution also should be raised when distinct effects of antagonists are shown in rodents and *homo sapiens*.

**FIGURE 10:**
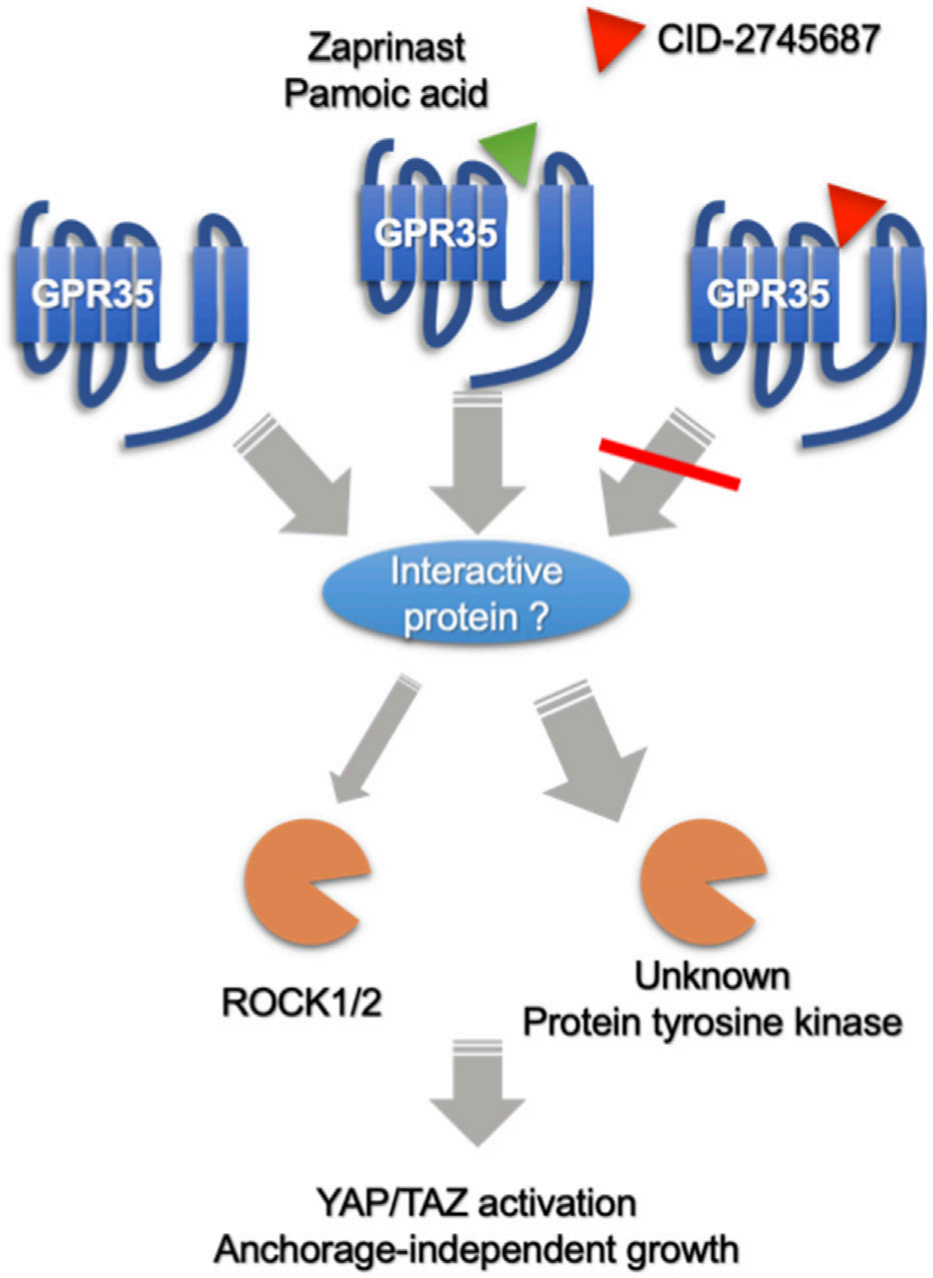
Speculative summary on the mechanism of GPR35 and its antagonist on CRC cells. GPR35 expression promotes anchorage-independent growth of CRC cells by enhancing YAP/TAZ activation partly through Rho-GTPase/ROCK1/2 axis, where other unknown protein tyrosine kinase may also participate. Furthermore, GPR35 activity modulates YAP/TAZ activity by associating with other proteins or receptors in an agonist-independent manner. Antagonists, like CID-2745687 and ML145, perturb GPR35 to association with interacting protein, exhibiting an inhibitory effect on YAP/TAZ activity. On the other hand, Agonists, like zaprinast and pamoic acid, do not further promote GPR35-mediated YAP/TAZ activity but can compete with CID-2745687, attenuating the inhibitory effect of CID-2745687.

GPCR family members regulated the Hippo pathway through G proteins-Rho GTPase-actin cytoskeleton signaling axis (Yu et al., 2012). Interestingly, GPR35 is distinctive from other GPCRs. Firstly, agonists did not promote YAP/TAZ activity; secondly, Rho-GTPase only partly contributed to GPR35-regulated YAP/TAZ activity; lastly, applying Gi/o inhibitor pertussis toxin or the knock-down of G13, Gi1, and β-arrestin2, mostly acknowledged downstream signal transducers for GPR35 (Jenkins et al., 2011; De Giovanni et al., 2022), showed no apparent inhibitory effects (Supplementary Figures S7, S8). However, the remaining expression of these shRNA knocked-down proteins could be sufficient to transduce GPR35 activation. In addition, Proto-oncogene tyrosine-protein kinase Src was implicated in GPR35 signaling (Schneditz et al., 2019), which was also reported to regulate YAP/TAZ activity (Sun et al., 2018). Nevertheless, dasatinib, a specific inhibitor of the Src kinase family, did not consistently regulate YAP/TAZ in GPR35 overexpressed cells (Supplementary Figure S9). Therefore, we speculated that GPR35 interacts with other receptors or protein tyrosine kinases, exhibiting constitutive activity on YAP/TAZ. Conceptionally, CID works by interrupting the GPR35 to interact with others (Figure 10). Finding the predominant molecule interacting with GPR35 in mediating YAP/TAZ would be significant work in the future.

The finding that GPR35 promoted YAP/TAZ activity makes it a very appealing target for anti-CRC therapy. Although YAP/TAZ was considered entirely dispensable for intestinal homeostasis (Hageman et al., 2020), it was found to be positively correlated with poor prognosis in CRC patients (Yuen et al., 2013) and required for metastatic colonization of CRC cells (Heinz et al., 2022). The dispensability of YAP/TAZ in normal intestinal homeostasis and its potent proliferative and pro-survival actions, when overexpressed in CRC, makes it an attractive therapeutic target (Zhou et al., 2011). However, YAP/TAZ is universally expressed in many other tissues and plays profound roles. Therefore, targeting YAP/TAZ is not a rational therapeutic strategy. GPR35 is expressed relatively highly in the gastrointestinal tract (Kaya et al., 2021), making GPR35 antagonists promising agents targeting YAP/TAZ overexpression or hyperactivation in CRC. However, antagonists only inhibited GPR35-mediated YAP/TAZ activity, meaning antagonists cannot control YAP/TAZ hyperactivation or overexpression attributed to other upstream regulators. Therefore, clinical research is required to determine to what extent YAP/TAZ activity in CRC cells is associated with GPR35 activity.

In summary, we discover a function of agonist-independent constitutive GPR35 activity that promotes YAP/TAZ activity and assists cancer cell growth under detachment. Furthermore, these findings unveiled an underlying mechanism of GPR35 in CRC progression. Finally, we highlighted the importance of developing GPR35 antagonists by suggesting their promising therapeutic potency in CRC.

## Data Availability

The original contributions presented in the study are included in the article/Supplementary Material, further inquiries can be directed to the corresponding author.
