# Supplementary material for: GPR35 antagonist CID-2745687 attenuates anchorage-independent cell growth by inhibiting YAP/TAZ activity in colorectal cancer cells
Source: Front Pharmacol. 2023 Apr 11;14:1126119. doi: 10.3389/fphar.2023.1126119 (PMC10126512; doi:10.3389/fphar.2023.1126119)
Supplement: Supplementary file 1 [file DataSheet1.PDF]

Supplementary Material

GPR35 antagonist CID-2745687 attenuates anchorage-independent cell growth by inhibiting YAP/TAZ activity in colorectal cancer cells.

Wuxiyar Otkur[1], Xiaolong Liu[1], Huan chen[1], Siyi Li[1], Ting Ling[1], Hanchen lin[1], Renyu yang[1], Tian xia[1], Huan Qi[1], Hai-long Piao[1*]

*** Correspondence:** Hai-long Piao, hpiao@dicp.ac.cn

1.1 Supplementary Figures

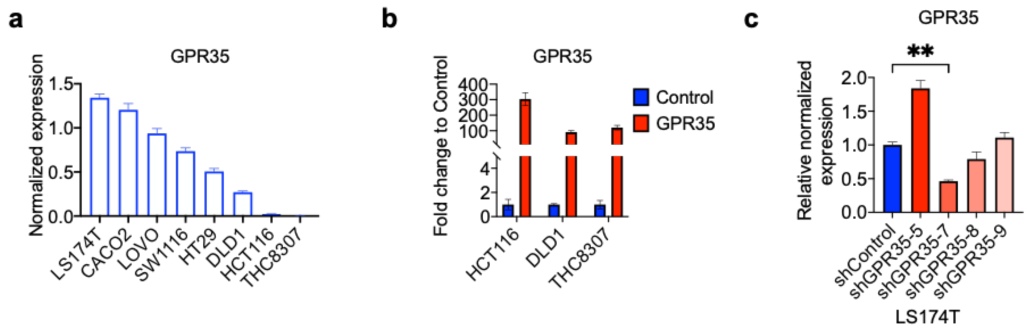

Supplementary Figure 1. Expression of GPR35 in colorectal cancer cell line and establishment of GPR35 overexpressed or knocked-down cell lines. (a) Normalized mRNA expression of *GPR35* in different CRC cell lines. **(b)** mRNA expression of *GPR35* in establish cell lines. **(c)** Verification of GPR35 targeting shRNA in LS174T cells by examining mRNA expression of *GPR35*.

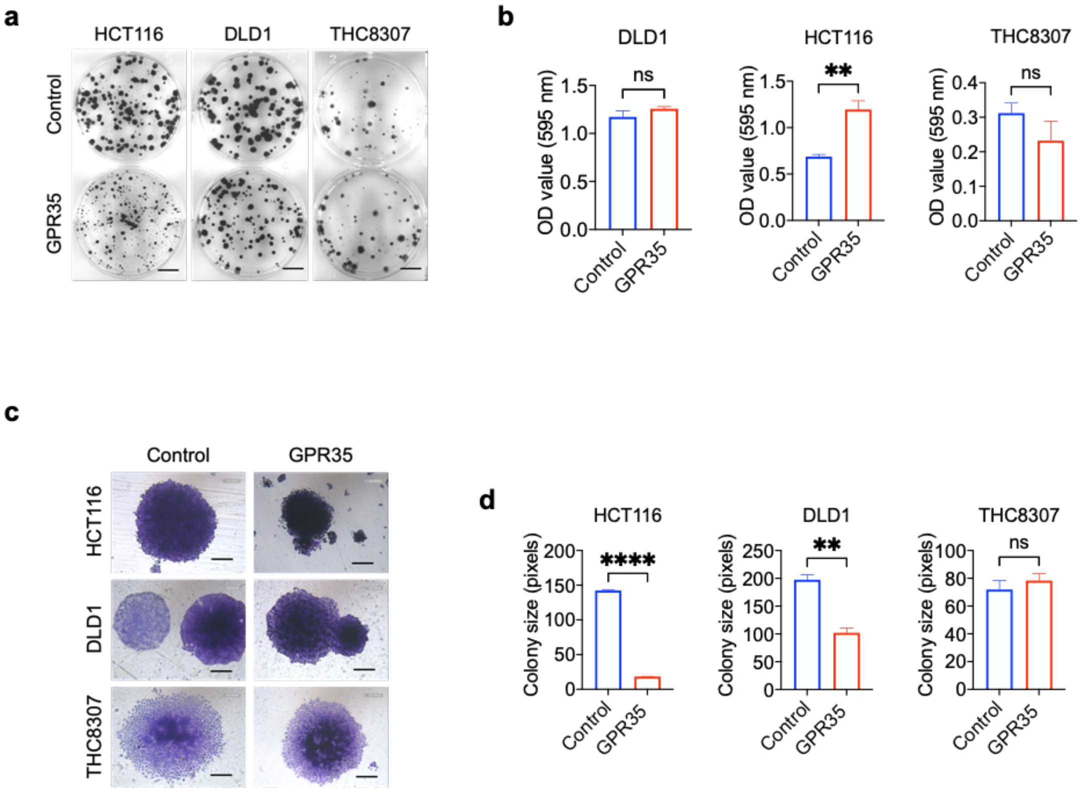

Supplementary Figure 2. GPR35-overexpressed DLD1 and HCT116 formed dense crowd-like colonies. (a) Representative image of the 2D colonies. Cells were seed at the density of 200 cells per wells. After 14 days of culture, cells were stained with crystal violet and observed with microscope. Scale bars: 1cm. (**b**) Quantification data of crystal violet staining. (**c**) Representative microscopic images of colonies. Scale bars: 0.1 mm. (**d**) Quantification data of colony sizes.

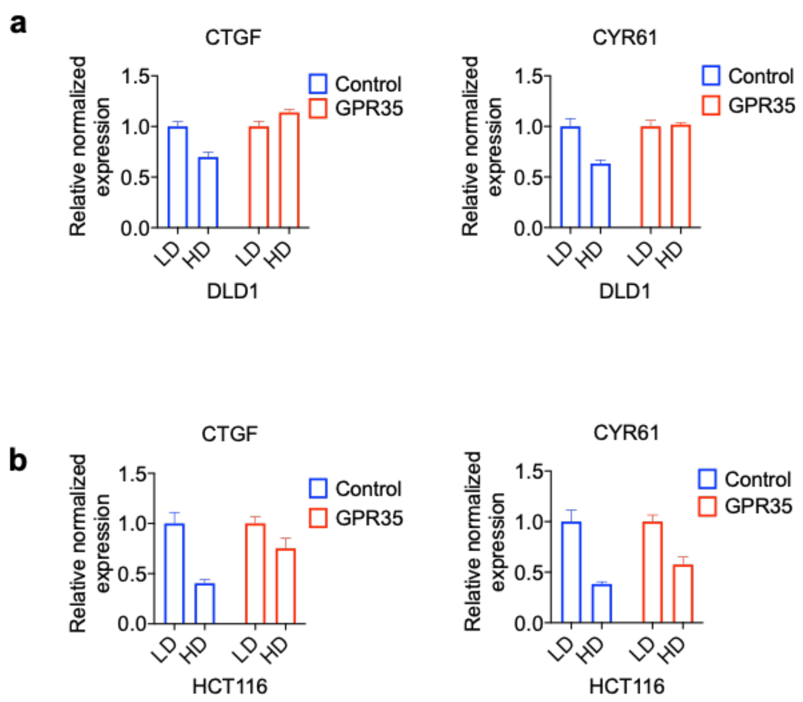

Supplementary Figure 3. GPR35 attenuated the decrease of YAP/TAZ activity by high cell density in DLD1 and HCT116. 1×10^6 cells were seeded in 10 cm dish as the low-density culture condition (LD), and 1×10^6 cells were seeded in 6-well plates as the high-density culture condition (HD). After 24 h of culture, cells were harvested for PCR assay. (**a**) YAP/TAZ target genes expression in established DLD1 cell lines cultured in different cell density conditions. **(b)** For HCT116 cell lines.

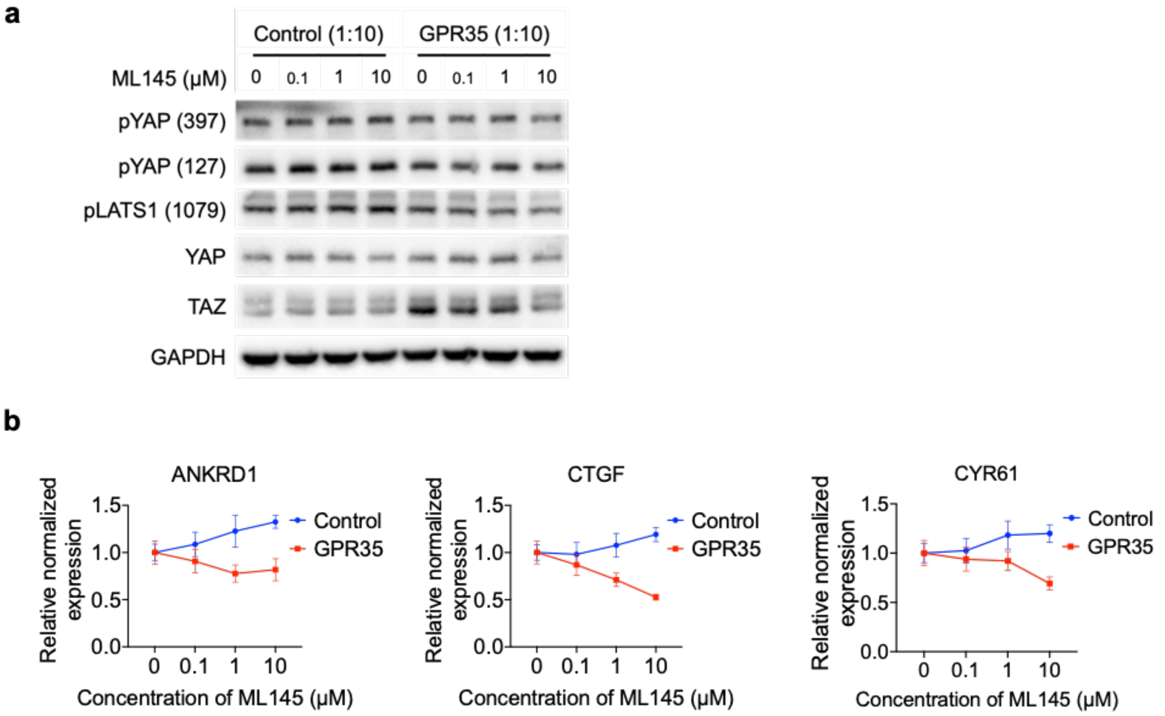

Supplementary Figure 4. GPR35 antagonists ML145 inhibited YAP/TAZ activity in GPR35 overexpressed cells. 293FT cells were infected with GPR35 expression lentivirus at different ratio of 1:10 for 24 h, and the different concentrations of antagonist were added directly to the medium. **(a)** Western blot analyzing expression and phosphorylation of YAP, TAZ and LATS1 after treatment with different concentrations of ML145. **(b)** mRNA expression of YAP/TAZ target genes after treatment of ML145

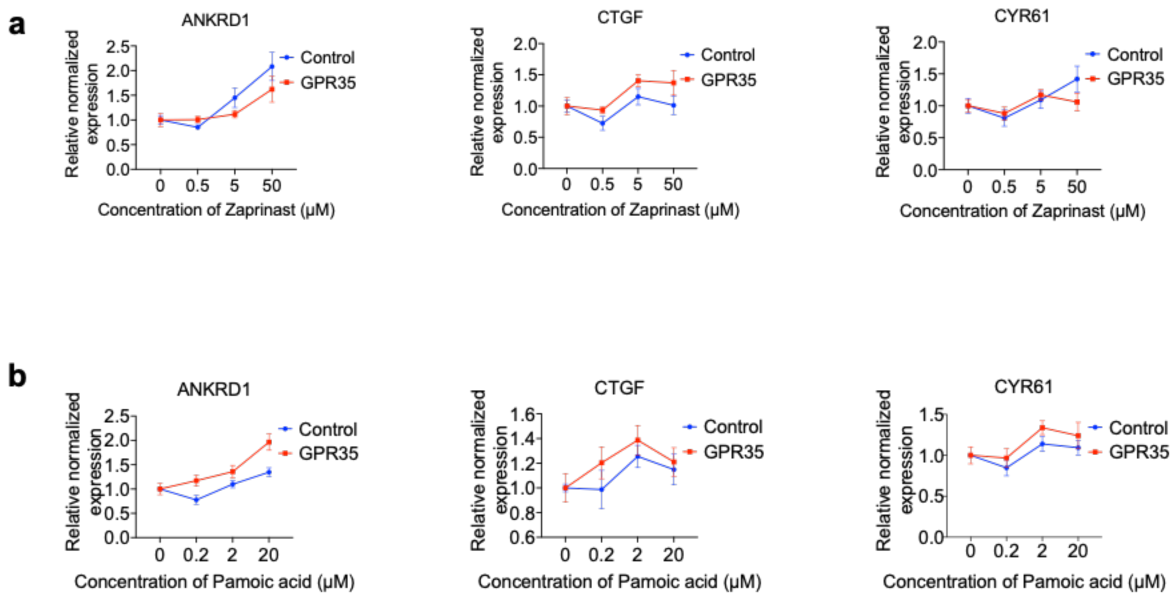

Supplementary figure 5. GPR35 agonists did not promote YAP/TAZ activity in GPR35 expressed 293FT cells. 293FT cells were infected with GPR35 expression lentivirus at different ratio of 1:20 for 24 h, and different concentration of agonists were added directly to the medium. **(a)** mRNA expression of YAP/TAZ target genes after treatment with different concentration of zaprinast. (**b**) mRNA expression of YAP/TAZ target genes after treatment with different concentration of pamoic acid.

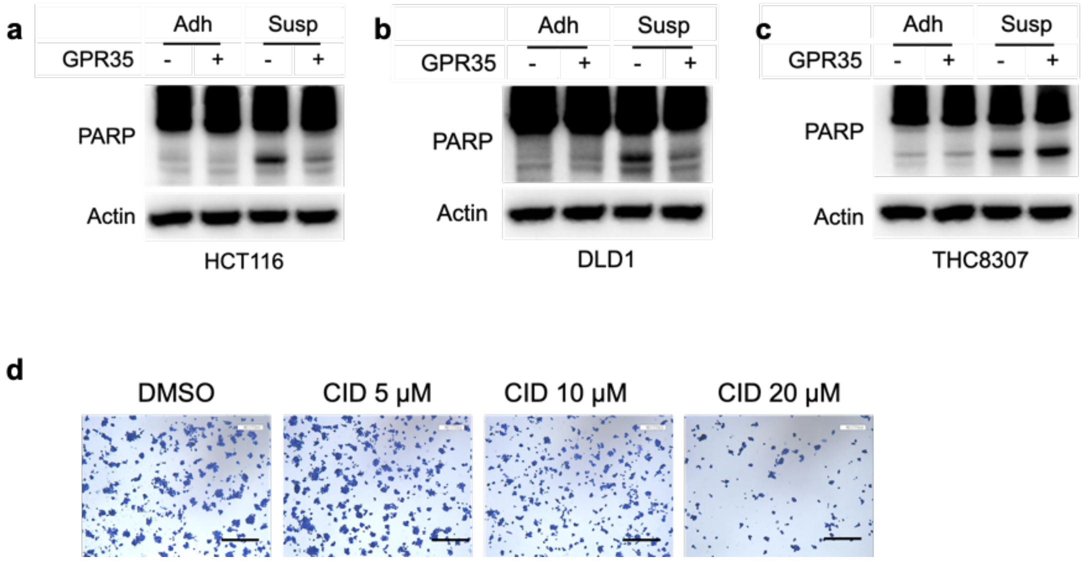

Supplementary Figure 6. GPR35 overexpression attenuated anoikis and CID-2745687 promoted anoikis in colorectal cancer cells. Cells were culture in unattached conditions on 1% agarose-coated plates (Susp), and cells were culture in plastic plates in cell adhesion conditions (Adh) as the control. Cells were harvested for western blot assay after 48 h of culture. **(a)** Western blot of PARP cleavage. **(b)** Microscopic images of crystal violet staining of re-adherent LS174T cells. LS174T cells were cultured in unattached conditions on 1% agarose-coated plates with different concentrations of CID for 24 h. Cells were collected, trypsinized, transferred to cell culture plates, and further cultured for 24 for re-adhesion. Scale bar: 0.1 mm.

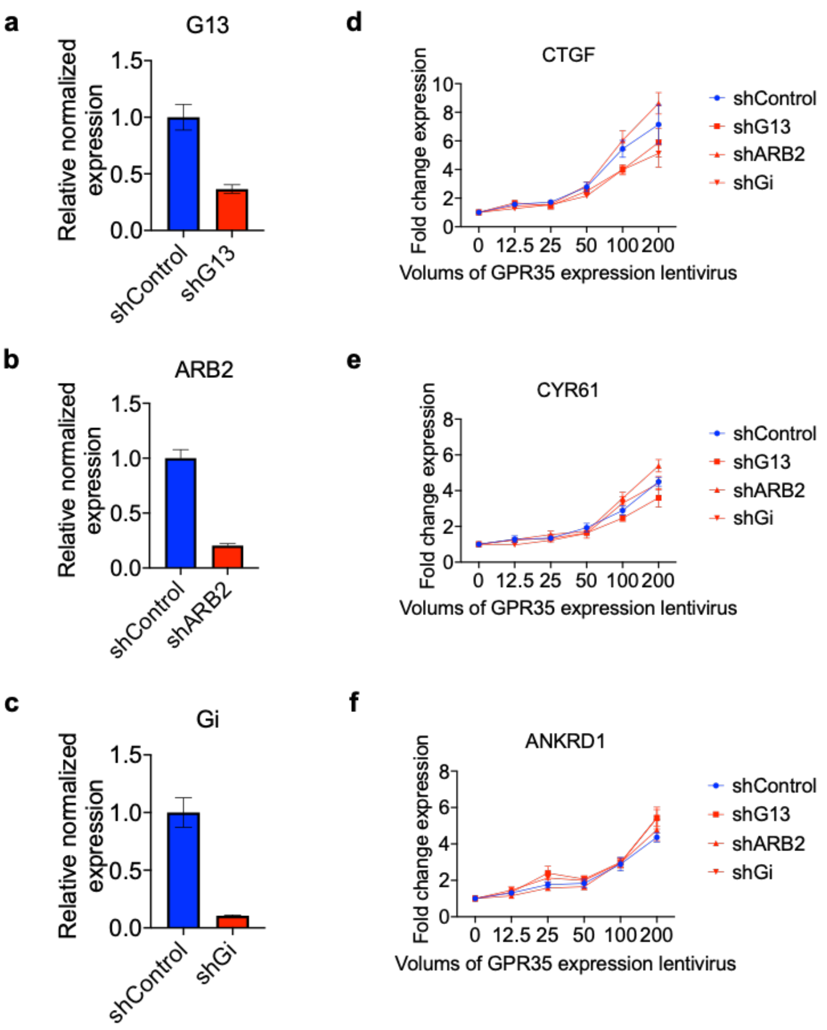

Supplementary Figure 7. Knock-down of G13, Gi, and β-Arrestin2 did not significantly inhibit GPR35 promoted YAP/TAZ activity. (a-c) Real-time PCR examining the knock-down efficiency of shRNA in 293FT cells; (**a**) for knock-down of G13; (**b**) for knock-down of β-arrestin2 (ARB2); (**c**) for knock-down of Gi. **(d-f)** mRNA expression of *CTGF*, *CYR61* and *ANKRD1*. Establish cell lines were infected with indicated lentivirus for 48h, and harvested for real-time PCR assay.

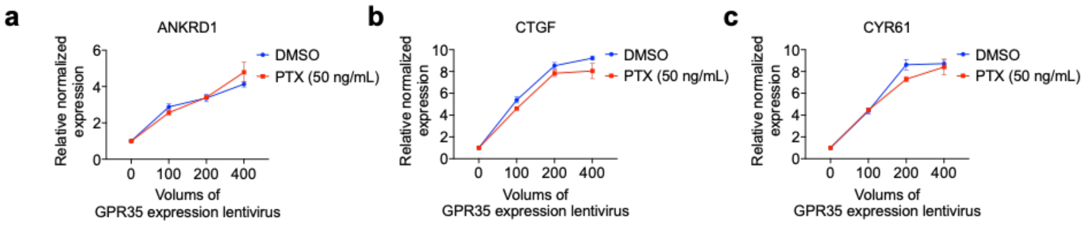

Supplementary figure 8. Gi/o inhibitor pertussis toxin did not block GPR35-mediated YAP/TAZ activity. (a-c) mRNA expression of YAP/TAZ target genes. 293FT cells were infected with different volumes of GPR35 expression lentivirus for 24h, and 50 ng/mL PTX was added directly to the medium for another 24 h. Then cells were harvested for PCR assay.

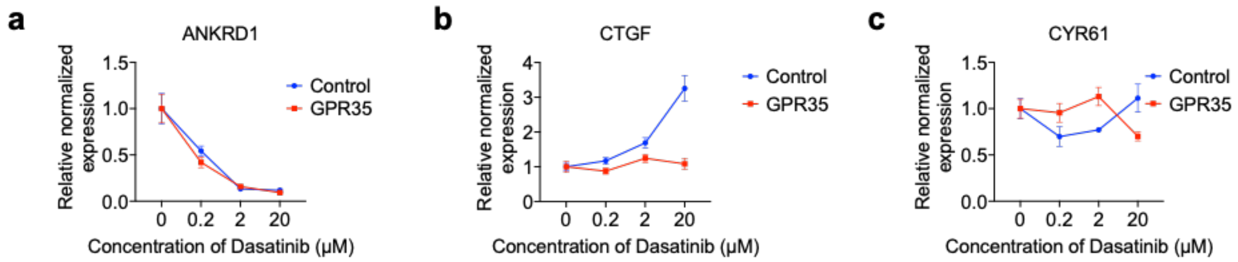

Supplementary Figure 9. Src kinase inhibitor dasatinib did not inhibit YAP/TAZ target genes expression promoted by GPR35 overexpression. 293FT cells were infected with GPR35 expression lentivirus containing medium at different ratio of 1:10 for 24 h and different concentrations of dasatinib were added directly to the medium. **(a-c)** The mRNA expression level of YAP/TAZ target genes.

frontiers
